# Complete *de novo* assembly of *Wolbachia* endosymbiont of contemporary *Drosophila simulans* using long-read genome sequencing

**DOI:** 10.1128/mra.00992-25

**Published:** 2026-04-30

**Authors:** Jodie Jacobs, Alexandra Lum, Elyse Mina, Camryn N. Morey, Darren D. Lee, Emry Gutierrez, Jonah Dionisio, Cade Mirchandani, Luke Sylvester, Anne Nakamoto, Hailey Loucks, Ciara Wanket, Ariana Cisneros, Alessandro Calicchio, Alexis N. Enstrom, Camille Headrick, Faith Okamoto, Harrison D. Heath, Kseniya Malukhina, Petria Russell, Sagorika Nag, Thomas Gillespie, William Sobolewski, Zia Truong, Shelbi L. Russell

**Affiliations:** 1 Biomolecular Engineering Department, University of California 8787, Santa Cruz, California, USA; 2 Genomics Institute at the University of California 624481, Santa Cruz, California, USA; 3 Ecology and Evolutionary Biology Department, University of California 8787, Santa Cruz, California, USA; University of Maryland School of Medicine, Baltimore, Maryland, USA

**Keywords:** genome, symbiosis, *Wolbachia*, *Drosophila*, evolution, evolutionary biology, biological control, ecology

## Abstract

We present a contemporary high-quality, complete *de novo* assembly of *Wolbachia pipientis* (*w*Ri Merrill 23, OZ411647), an alphaproteobacterial endosymbiont of *Drosophila simulans (D. simulans)*. This assembly was generated using long-read sequencing of *w*Ri-infected *D. simulans* embryos collected from Merrill College at the University of California, Santa Cruz, in October 2023.

## ANNOUNCEMENT

*Wolbachia pipientis* infects diverse arthropods and nematodes, manipulating host phenotypes through cytoplasmic incompatibility (CI), male killing, and fertility rescue (1, 2). The Riverside strain (*w*Ri) was first identified in California *Drosophila simulans (D. simulans)* in the 1980s (3) and rapidly spread statewide due to exceptionally strong CI (4). Despite its significance (5), the only reference genome reflects *w*Ri present in 1984 (3, 6). Here, we present a complete *de novo w*Ri genome assembly from contemporary *D. simulans* collected at the UC Santa Cruz Alan Chadwick Garden in Merrill College in October 2023.

To generate a contemporary *w*Ri genome assembly, we collected wild *D. simulans* flies, established isofemale lines, and sequenced *w*Ri-infected embryos. We established isofemale lines by deploying banana-baited bottles for ~5 days and collecting gravid females onto white food medium. After offspring eclosed, species identity was confirmed by phenotyping males and by PCR using silf-F/R primers to distinguish *D. simulans* from *D. melanogaster* (7) and wsp_1F/592R primers to confirm *w*Ri identity (8). We extracted DNA from *w*Ri-infected embryos using the Wizard HMW DNA Extraction Kit (Promega) without shearing and prepared libraries with the Native Barcoding Kit V14 (SQK-NBD114-24) without size selection. We sequenced these libraries on the Nanopore MinION Mk1B with an R10 flow cell (FLO-MIN-114) and MinKNOW v23.07.8 with adaptive sampling (fast model) to deplete *D. simulans* reads (GCF_016746395.2) yielding 4.26M reads, (N50 749 bp, 20 h) that were basecalled with Dorado (v0.7.3, hac model, --min-qscore 10). After filtering for host-free reads >3 kb (93K reads remaining, N50 10.5 kb), we assembled the *w*Ri genome using Flye (9) following the methods described by Jacobs and Nakamoto *et al*. (10), yielding a 1.26 Mb circular assembly with 30× coverage. Flye determined circularization (4,000 bp minimum overlap), the genome was not rotated.

**Fig 1:**
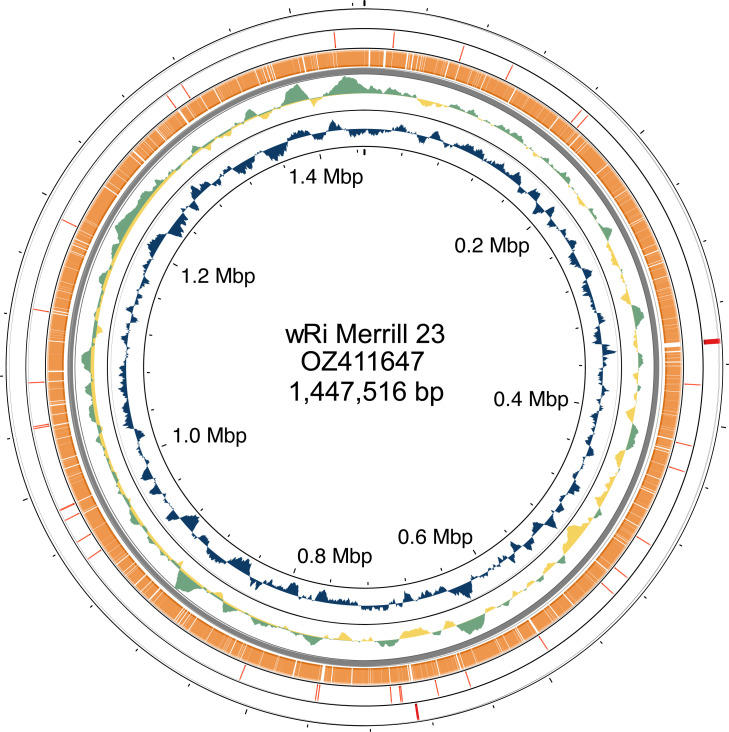
*Wolbachia w*Ri genome map. Concentric circles show (outer to inner) rRNA genes (red), tRNA genes (red), coding sequences (orange), GC skew (green/yellow for high/low), and GC content (blue), with GC metrics plotted as deviations from the genome-wide average.

To polish the assembly, we generated Illumina short-read whole-genome sequencing data from whole *w*Ri-infected *D. simulans* flies (Merrill 23 stocks). Illumina libraries were prepared using a Tn5-based tagmentation protocol (11). The Tn5 enzyme (Tn5R27S, E54K, and L372P) was obtained from QB3 MacroLab (University of California, Berkeley). Tagmentation simultaneously fragmented genomic DNA and ligated adapter sequences using custom oligos (Tn5-A: FC-121-1030, Tn5-B: FC-121-1031, and Tn5-rev; IDT). Libraries were indexed and amplified using the Nextera XT Index Kit v2 (i5/i7 adapters, Illumina) with KAPA HiFi (Roche) and sequenced on a NovaSeqX Plus (2 × 150 bp). Reads were trimmed to remove sequencing adapters and low-quality regions using fastp v1.0.1 (11) before polishing the assembly with Pilon v1.24 (12) (849K reads). We assessed the quality of the polished assembly with BUSCO v5.7.0 (13) (rickettsiales_odb10), achieving 99.2% completeness; annotated the assembly with Prokka v1.1.1 (14) (kingdom:bacteria) (Table 1); and calculated and visualized GC content and GC skew with Proksee v1.1.2 (15) (Fig. 1). Default parameters were used unless otherwise specified.

**TABLE 1:** Annotation summary statistics

wRi Merrill 23 annotation summary	
Annotation pipeline	Prokka v1.1.1
Annotation method	kingdom:bacteria
Length (bp)	1,447,516
GC Content	35.21%
Genes (total)	1,392
CDSs (total)	1,392
Genes (RNA)	37
rRNAs	1, 1, 1 (5S, 16S, 23S)
tRNAs	34
ncRNAs	0
Pseudogenes (total)	0

## Data Availability

Raw sequencing reads, ERX15644803 (Illumina) and ERX15644805 (ONT) and the assembled genome (OZ411647) are available under BioProject accession number PRJEB107302. Assembly pipeline: https://github.com/jodiejacobs/Jacobs_et_al_2026_de_novo_wRi_merrill_23_assembly.
